# “The Early Specialised Bird Catches the Worm!” – A Specialised Sampling Model in the Development of Football Talents

**DOI:** 10.3389/fpsyg.2018.00188

**Published:** 2018-02-21

**Authors:** Roland Sieghartsleitner, Claudia Zuber, Marc Zibung, Achim Conzelmann

**Affiliations:** Institute of Sport Science, University of Bern, Bern, Switzerland

**Keywords:** talent development, early engagement, football, specialisation, diversification/sampling, specialised sampling model, person-oriented approach

## Abstract

Characteristics of learning activities in early sport participation play a key role in the development of the sporting talent. Therefore, pathways of specialisation or diversification/sampling are as well debated as the implementation of practice- or play-oriented activities. The related issues are currently perceived as a two-dimensional construct of *domain specificity* and *performance orientation*. In this context, it has been shown that early specialisation, with experiences in practice and play, has led to Swiss junior national team football players reaching higher success levels as adults. This study aimed to examine whether a similar approach improves chances of even being selected for junior national teams from a broader sample. Hence, 294 youth players answered retrospective questionnaires on their early sport participation when entering the Swiss football talent development programme. Using the person-oriented Linking of Clusters after removal of a Residue (LICUR) method, volumes of in-club practice, free play and activities besides football until 12 years of age were analysed along with age at initial club participation. According to the results, clusters of *Football enthusiasts* (*p* = 0.01) with the most free play and above average in-club practice and *Club players* (*p* = 0.02) with the most in-club practice and average free play had a greater chance of reaching junior national team level. Thus, high levels of domain-specific activities seem to increase the chances of junior national team participation. Furthermore, the most successful constellation (*Football enthusiasts*) may illustrate the relevance of domain-specific diversity, induced by several types of practice and play. In line with previous studies, specialising in football and sampling different experiences within this specific domain seems to be the most promising pathway. Therefore, we argue that the optimal model for the development of football talents is a *specialised sampling model*.

## Introduction

For economic and prestige reasons, football organisations try to develop outstanding players (Relvas et al., 2010; Grix and Carmichael, 2012). To succeed in this aim, clubs and national federations have to think twofold. First, the most talented players with the potential to become elite athletes have to be identified at the right time. Second, optimising the learning environment is then crucial for developing these players (Williams and Reilly, 2000). In the search for this optimisation, several athlete development models have emerged from research and been adapted and implemented by professional sport organisations (Bruner et al., 2010; Côté and Vierimaa, 2014). Among other factors, practice contributes highly to these development models on the pathway to expertise (Baker and Horton, 2004; Rees et al., 2016). However, apart from a broad consensus statement on the essential role of practice from an early age, several open questions regarding its characteristics within youth sports participation are vigorously debated in the field of sport science (Côté et al., 2007). For that reason, policy makers in clubs and federations may struggle in designing evidence-based structures within talent development in football, demonstrating a need for further research on the parameters of practice for child athletes (Mountjoy et al., 2008).

Out of the open questions on the properties of learning activities in youth sports participation, two well-known ones deal with the subsequently introduced dimensions *domain specificity* and *performance orientation* (Storm et al., 2012; Coutinho et al., 2016b). We perceive domain specificity as the degree of congruence in biomechanical, physiological and psychological characteristics between learning activities and the primary sport domain of an athlete. Thus, it represents a major issue in the common *specialise or sample* debate (Côté et al., 2009), i.e., whether young talents should focus on a single sport-specific domain early, or try to build a foundation with broad and different experiences from several kinds of sports. In general, the former is known as *specialisation* (high value of domain specificity), the latter as *diversification/sampling* (low value of domain specificity). The second dimension of interest, the performance orientation, is perceived as a summation of a few structural characteristics of learning activities. By a combination of various degrees of goal setting, monitoring and correction (Côté et al., 2003), each learning activity receives a certain degree of performance orientation. The literature mainly differentiates between *practice* as a highly structured, coach-led activity (high value of performance orientation) and *play*, which represents a fun-oriented learning activity without supervision (low value of performance orientation; Côté et al., 2007).

**FIGURE 1:**
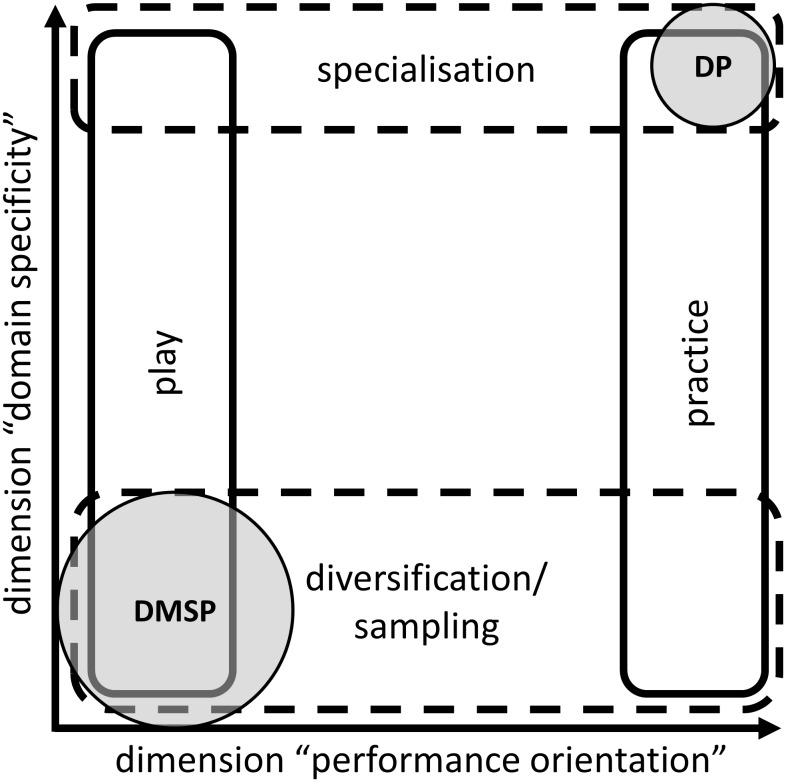
The two-dimensional construct of domain specificity and performance orientation and each of its dichotomous counterparts. Deliberate practice framework (DP) and the elite performance through sampling pathway from the development model of sports participation (DMSP) are perceived as intersections of those dimensions.

Both presented dimensions play key roles in the development of the sporting talent (Bridge and Toms, 2013). As Coutinho et al. (2016b) named a sometimes neglected interaction between domain specificity and performance orientation, they are currently described as a two-dimensional construct of characteristics of learning activities. Though both dimensions seem to be a continuum with a broad spectrum of possibilities, many researchers reduce each case to the previously mentioned dichotomous counterparts of specialisation vs. diversification/sampling and practice vs. play (Baker et al., 2009; cf. **Figure 1**).

Côté and Erickson, 2015 recently reconceptualised the idea of diversification/sampling. They pointed out that one can specialise in a single sport-specific domain and sample through different experiences of the same sport domain (e.g., in case of football: playing and practising with your club, playing beach soccer with friends, playing in a park just right after school, and playing on your own). Although this perception would enable a coexistence of concurrent specialising and sampling, the literature on domain specificity is still dominated by a controversial, polarised debate.

The origin for the dichotomising of domain specificity and performance orientation is obviously the opposite profile and huge influence of two well-established concepts for developing expertise in sports, the deliberate practice framework (Ericsson et al., 1993; Ericsson, 2008; Baker and Young, 2014) and the development model of sport participation (Côté, 1999; Côté et al., 2007; Côté and Vierimaa, 2014). According to Ford et al. (2015), deliberate practice is based on the monotonic benefits assumption. This stands for a linear relationship between the amount of highly effortful and structured activity with the explicit goal of improving performance, and the acquired performance in a specific task. Naturally this means that each learning activity may be carefully monitored, with a focus on immediate correction (Côté et al., 2003). In summary, the deliberate practice view can be related to the idea of maximising domain specificity (specialisation) and performance orientation (practice) from an early age. In contrast, although the development model of sport participation accepts and integrates the deliberate practice framework as one possible solution to enabling elite performance, it also creates a multi-staged opposite position (elite performance through sampling) with initial sampling years up to 12 years of age. This view recommends an early involvement in several sports in combination with fun-oriented and loosely monitored learning activities as the preferable way to expertise (Côté, 1999; Côté et al., 2003, 2007). In other words, elite performance through sampling represents a minimum of domain specificity (diversification/sampling) and performance orientation (play) in the early stage of development, followed by an increasing amount of deliberate practice from 12 years on (Côté et al., 2007).

Researchers have hypothesised some mechanisms to explain the learning effects according to low values of domain specificity and performance orientation in youth learning activities. First, some researchers have explored possible transfer effects throughout different sports from diversification/sampling (Baker et al., 2009; Fransen et al., 2012). Second, others have assumed an increase in intrinsic motivation (Hendry et al., 2014; Vink et al., 2015) or tactical performance (Greco et al., 2010; Memmert et al., 2010) from playful activity. Finally, if these conjunctions of diversification/sampling and play are not able to compensate or even overshoot the accumulated loss in domain-specific and performance-oriented practice volume from an early specialisation, the elite performance through sampling advocates still claim that it may lead to the same senior skill level without negative consequences (Côté and Hancock, 2015). These possible risks of an early specialisation include, for example, damage to health (Law et al., 2007; Jayanthi et al., 2013; Difiori et al., 2014; Pasulka et al., 2017), higher incidence of dropout (Wall and Côté, 2007; Fraser-Thomas et al., 2008) and burnout (Strachan et al., 2009). A high degree of reservation and numerous warnings against early specialisation have resulted from these findings (Wiersma, 2000; Malina, 2010; Mostafavifar et al., 2013; Bergeron et al., 2015; Feeley et al., 2016; LaPrade et al., 2016; Read et al., 2016). Moreover, the effectiveness of specialised and performance-oriented practice has recently been critically scrutinised. According to Macnamara et al.’s (2016) meta-analysis, deliberate practice only explains 18% of the variance in sports performance, contrary to the monotonic benefits assumption. Overall, there is increasing criticism of specialisation and high performance-orientation within early sports participation, although their contribution to the development of expertise has been shown many times (Côté et al., 2003; Baker et al., 2003a, 2009; Baker and Young, 2014; Ford et al., 2015).

The inconsistency in the findings and positions according to this dichotomous debate supports the absence of a single solution for all sporting contexts (Suppiah et al., 2015). However, in the case of a single view on talent development in football, the distribution of learning activities shows a more consistent picture. Ford et al. (2009) offered the early engagement hypothesis, which provides a football-specific pathway to expertise. Consisting of an early entrance into the domain, an extensive amount of both, practice and play, and a substantially smaller amount of hours in sports besides football, this pathway has been recognised several times as a promising one (Helsen et al., 1998; Ford et al., 2009, 2012; Roca et al., 2012; Williams et al., 2012; Haugaasen et al., 2014). While finding similar characteristics in the allocation of learning activities, Ford and Williams (2012) and Hornig et al. (2016) claimed a more relevant influence of diversified learning activities besides football. However, all of these investigations may suffer from methodological problems, as there are difficulties in categorising different learning activities in team sports (Helsen et al., 1998). These problems notwithstanding, an early specialisation combined with activity types from different degrees of performance orientation obviously describe a promising pathway for talent development in football.

Beneath the characteristics of a specific sport domain, country- or culture-specific environments may also contribute to inconsistencies in the described debate (Baker et al., 2003b; Storm et al., 2012; Suppiah et al., 2015; Forsman et al., 2016). Although Ford et al. (2012) did find similarities between development activities of elite soccer players from different countries, Araújo et al. (2010) emphasised that specific environment constraints such as in Brazilian football (i.e., football played with adapted norms and rules in a variety of locations), may lead to specific pathways to expertise. In addition, Holt (2002) showed that talent development systems in football differed a great deal between Canada and England.

Overall, it may therefore be difficult to generate a viable answer to the appropriate configuration of domain specificity or performance orientation within youth sports participation from distinct sport domains or countries. There seems to be no *one-model-fits-all* approach, as “the details of the developmental route undertaken by a successful elite athlete largely depends on the nature of the sport, and the culture and context of the country” (Suppiah et al., 2015).

To shed further light on the current issue in the context of Swiss Football, Zibung and Conzelmann (2013) asked former Swiss junior national team (SJNT) players about their sport participation up to 12 years of age. Based on the multi-dimensional nature of the dataset and its context of developing human individuals, their data analysis followed a development-related perspective arising from the holistic and dynamic-interactionistic approaches of developmental science (Bergman and El-Khouri, 2003; Bergman and Andersson, 2010). These concepts question the existence of a single *General Linear Model* and the application of common variable-oriented methods (Bergman and Andersson, 2010). Hence, Zibung and Conzelmann (2013) used a person-oriented approach (Bergman and El-Khouri, 2003), which seems to be appropriate for issues of talent development by enabling the opportunity of non-linear interaction between single characteristics within each individual (Bergman and Andersson, 2010).

Using the person-oriented approach, Zibung and Conzelmann (2013) found results in alignment with the aforementioned early engagement hypothesis (Ford et al., 2009). High values of domain specificity consisting of both, practice and playful activities led to players reaching higher success levels as adults. Regarding the participants, one has to mention that SJNT players are already a highly selected population. At most, 2% of all registered football players in Switzerland have the chance to participate at that elite youth level (Romann and Fuchslocher, 2013), which becomes more and more relevant for reaching elite levels in adult football. Regarding late adolescence, participating in elite youth football development programmes (e.g., academies or junior national teams) has a significant impact on later participation in professional teams. For example, almost 90% of all German Bundesliga players (seasons 2009/2010 to 2011/2012) had been involved in a youth academy for at least one season (Güllich, 2014). Also, around 60% of German U19 national team participants become Bundesliga players and the same amount of Portuguese U17/18 national team players are selected for the senior national team later in their career (Barreiros et al., 2014; Güllich, 2014).

In addition to Zibung and Conzelmann’s (2013) study, which dealt with the pathway from elite youth football in late adolescence to adulthood, it would be interesting to know if similar distributions of learning activities in the initial phase of the sports career also boost the chances of even participating in elite youth football development programmes. For that reason, we investigated whether SJNT players had more domain-specific and performance-oriented experiences up to 12 years of age, than their less successful peers. Together with the aforementioned results, this may contribute to the relevant knowledge on domain specificity and performance orientation of learning activities within a talent development system in football.

## Materials and Methods

### Participants

The current investigation is part of the longitudinal study *talent selection and talent development in Swiss football* (Zibung et al., 2016; Zuber et al., 2016), which has involved collecting data from different dimensions to describe talent development holistically (e.g., motor performance, psychological aspects, and external support). The study followed a substantial number of players born in 1999 throughout the talent promoting system of the Swiss Football Association from the initial selection into regional squads at the U13 age group up to junior national team selections (until U18).

As is common in other federal talent development programmes, the promotion system of the Swiss Football Association follows the pyramidal *standard model of talent development* (Bailey and Collins, 2013; Gulbin et al., 2013; Güllich, 2014). Therefore, only some of the players were able to stay in the system over the whole period, while many became deselected on the way and others entered later. Consequently, the total sample of 294 participants represents a quite heterogeneous group according to their success in youth football. Some did not even make it to the regional squad (*n* = 54, Level 3, local players), others got at least one nomination for a SJNT (U15 to U18 national teams, *n* = 57, Level 1, national players). The remaining 183 players reached an intermediate level somewhere between regional squads and the SJNTs (Level 2, regional players). **Table 1** gives an overview of these youth football success levels.

**Table 1:** Description and distribution of the three levels of youth football success.

Label	Description	Definition	Frequency	Percentage
Level 1	Players at national level	At least one nomination for SJNT^*1*^ (U15 – U18)	57	19.4
Level 2	Players at regional level	Passed regional squad selection; no SJNT	183	62.2
Level 3	Players at local level	Failed regional squad selection	54	18.4
Total			294	100.0

*^1^SJNT, Swiss junior national team*.

This study was carried out in accordance with the recommendations of the Ethical Principles of Psychologists and Code of Conduct, Ethics Committee of the Faculty of Human Sciences of the University of Bern. All players and their legal representatives gave their written informed consent to participate.

### Operationalisation and Data Collection

To gain an insight into domain specificity and performance orientation within the early sport participation of the participants, the latter was operationalised through four variables: (1) *volume of organised in-club football practice*, (2) *volume of free play within football*, (3) *volume of sports activities besides football* and (4) *the age at initial football club participation*. All data were collected with retrospective questionnaires, which asked the participants to report their sport behaviour until the start of the longitudinal study at the U13 age group. The questionnaire was administered in paper-and-pencil format and sent by post. Thereby participants had the possibility to complete them together with their parents at 12.93 ± 1.32 (mean ± SD) years of participants’ age. According to the first language of parents, the questionnaire was presented in German or French language. **Table 2** shows an English translation of the questions and response types. Volumes of all sporting activities were collected by means of hours per week in each age category since the entrance into sports. Afterwards these values were summed up to a total number of hours up to 12 years of age.

**Table 2:** Presented questions and response types of the retrospective questionnaire.

Operating factor	Presented question(s)	Response type(s)
(1) Volume of organised in-club football practice	How much did you practice within the football club in an average week since the start of your career (time of practice, without games)?	Number and total duration (h) of sessions per week, values for each age category
(2) Volume of free play within football	How many hours did you spend with free football in an average week (e.g., with colleagues, friends, on your own…)?	Total duration (h) per week, value for each age category
(3) Volume of sports activities besides football^*1*^	Please tell us any sport domain (besides football) you practiced on a regular base. How much did you practice within the club in an average week (time of practice, without games)?	Sport domain, number and total duration (h) of sessions per week in each domain, values for each age category
	How many hours did you spend with any other sporting activity than club practice, free football and physical education in an average week?	Total duration (h) per week, value for each age category
(4) The age at initial football club participation	What was your age at initial football club participation (distinct from age at initial free football participation)?	Age category (y)

*^1^Sum of two presented questions*.

The in-club practice subsumed in this matter any learning activity within a football club (or with an instructor). In contrast to this, free play describes every football activity outside the club and without supervision. This difference in the organisational structure of the activities is connected to a different amount of performance orientation (Côté et al., 2003) and should therefore be adequate to investigate the influence of the latter on success levels within youth football.

Furthermore, the two football-specific activities together (in-club practice and free play) represent the amount of domain-specific activities in conjunction with the issue of specialisation or diversification/sampling. In addition, the age at initial football club participation was used as another essential representation of early specialisation (Ford and Williams, 2012; Williams et al., 2012; Zibung and Conzelmann, 2013; Hornig et al., 2016).

The opposite part of the three football-related variables in terms of domain specificity is represented by the sports activities besides football. As researchers have previously found low total amounts of diversified activities within comparable samples (Ford et al., 2012; Ford and Williams, 2012; Haugaasen et al., 2014; Hornig et al., 2016), any kind of sports activities besides football, regardless of their organisational structure or performance orientation, were included within this variable. The sole exception was physical education within school, as this amount is more or less the same for each participant until the completion of comprehensive school at 13 years of age.

All of the four presented variables have been used in prior investigations (Zibung and Conzelmann, 2013; Hopwood, 2015; Hornig et al., 2016) and trace back to the fundamental expertise work of Hodges and Starkes (1996), who dealt extensively with psychometric properties of retrospective questionnaires. Reliability and validity of such methods in general, and the use of similar variables as in the current investigation in particular, have been shown to be acceptable (Hodges and Starkes, 1996; Helsen et al., 1998; Ford et al., 2010a; Memmert et al., 2010). Hopwood (2015) names Pearson correlation values for validity and reliability of involvement in practice activities (hours per week or per year) from 0.59 to 0.97 and from 0.79 to 0.99 for age at initial participation in primary sport. Ropponen et al. (2001) were able to show an ICC of 0.81 for retest reliability of mean exercise hours per week after 5 years within structured interviews.

### Data Analysis

Regarding various concepts of data analysing, there are substantial differences in dealing with multidimensional data of developing individuals. As already introduced, a person-oriented approach seems to be appropriate for such analysis (Bergman et al., 2003; Zibung and Conzelmann, 2013). This approach focuses on the individual explicitly and searches for promising non-linear patterns of a set of several variables within persons. These sets of interacting variables are referred to as *subsystems* of the whole individual, whilst the single variables building up the subsystems are known as *operating factors* (Bergman et al., 2003).

The person-oriented Linking of Clusters after removal of a Residue (LICUR) method (Bergman et al., 2003) was used to analyse the early sport participation in the current investigation. All of its subsequently described statistical procedures were carried out using the statistical package SLEIPNER (Bergman and El-Khouri, 2002) and followed the recommendations for person-oriented studies (Bergman et al., 2003; Bergman and El-Khouri, 2003).

An initial analysis of residues using the *Residue module* of SLEIPNER led to the exclusion of four cases with unique constellations of the operating factors, as their Euclidean distance to each of the other cases exceeded the *T* = 0.8 threshold value for *z*-standardised data. As the number of identified residues was below 3% of the whole sample, this part of data processing seems to be plausible in terms of content. For the subsequent cluster analysis (*Cluster module*), the Ward procedure with squared Euclidean distance was used. The determination of the best cluster solution was guided by content as well as by statistical criteria. Both, the elbow criterion and the Mojena stopping rule, with a threshold of 3.0 (Mojena, 1977), were used. Afterwards a partitioning cluster analysis (*k*-means method; *Relocate module*) was carried out to optimise the homogeneity within each cluster. Finally, the *Exacon modul*e was used to execute a transition analysis. The number of transitions from each cluster to the three levels of youth football success (cf. **Table 1**) was counted and tested for significance using Fisher’s exact test, with a hypergeometric distribution (*p* < 0.05). The amount of transitions was represented as a multiple of the expected value and expressed using odds ratios (*OR* = 1.0 as the expected value; *OR* < 1.0 means less and *OR* > 1.0 more transitions than expected by chance).

## Results

**Table 3** gives an overview of the total number of accumulated hours of activities during early sport participation up to 12 years of age, across specific clusters and throughout the entire sample. On average players completed a substantial amount of domain-specific in-club practice (1128 h). However, this accounts for only 22.5% of the total hours of all learning activities together. The players spent nearly twice as much time in free football play without supervision (2058 h, 41.0%) as in the club. Following from the broad inclusion of any kind of other activities, the sports activities besides football contributed to a remarkable extent to the early sport participation of the participants (1837 h, 36.6%). The initial involvement in organised football took place at an average age of 6.3 years and only five players in the whole sample entered their first football club later than 9 years of age.

**Table 3:** Descriptive statistics for the early sport participation (up to 12 years of age).

		*Early sport participation*					
		**In-club practice**		**Free play**		**Sports activities besides football**		**Age at initial club participation**	
		**(hours)**		**(hours)**		**(hours)**		**(years)**	
		***M***	***SD***	***M***	***SD***	***M***	***SD***	***M***	***SD***

Overall	(*n* = 290)^*1*^	1127.9	355.0	2058.3	1055.4	1836.7	1060.2	6.3	1.3
Cluster 1	(*n* = 25)	1304.2	269.7	4257.8	1404.4	1968.5	830.4	5.5	0.7
Cluster 2	(*n* = 56)	1602.4	229.8	1988.5	630.5	1736.9	821.9	5.4	0.9
Cluster 3	(*n* = 106)	1071.5	208.5	1694.9	633.4	1360.4	673.6	5.9	0.9
Cluster 4	(*n* = 42)	1091.1	217.2	2359.2	726.6	3592.7	863.5	6.4	0.7
Cluster 5	(*n* = 61)	743.2	202.6	1645.2	811.8	1493.0	721.0	7.9	1.2

*^1^Note that 4 cases were removed by residual analysis*.

**FIGURE 2:**
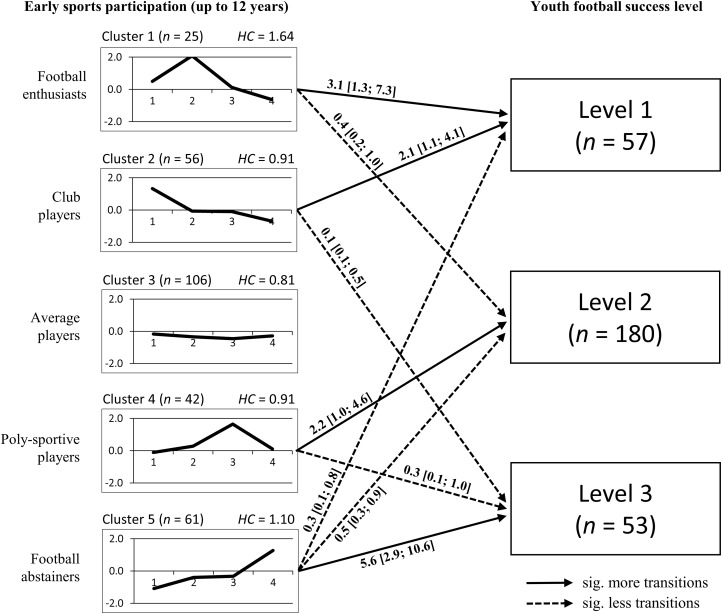
Profiles of *z*-scores of the five clusters and transitions to youth football success levels (EESS = 52.1%; weighted HC_mean_ = 0.97; SC = 0.52). Operating factors: 1 = hours of in-club football practice; 2 = hours of free play within football; 3 = hours of sports activities besides football; 4 = age at initial football club participation; Success levels: 1 = national players; 2 = regional players; 3 = local players; numbers for significant more (*OR* > 1.0) or less (*OR* < 1.0) transitions are expressed as odds ratios and 95% confidence intervals.

The cluster analysis extracted a five-pattern solution (cf. **Table 3** and **Figure 2**) and displayed an explained error sum of squares (EESS) of 52.1% after partitioning. Therefore it did not quite meet the desirable 2/3 criterion (Bergman et al., 2003). On the other hand, the total weighted mean of the homogeneity coefficients over all clusters reached a sufficient value (weighted HC_mean_ = 0.97) and the silhouette coefficient (SC = 0.52) illustrated that a reasonable structure was found in the analysed data (Kaufmann and Rousseuw, 1990; Vargha et al., 2015, 2016). Therefore, two out of three quality coefficients for the cluster analysis indicated an acceptable pattern solution.

According to the cluster profiles, cluster 1 was basically characterised by an extensive amount of free play within football (4258 h), which was around two standard deviations higher than average. In combination with a slightly increased amount of in-club practice (1304 h), which goes along with the early start of careers in football clubs at 5.5 years, these *Football enthusiasts* (*n* = 25) showed the highest number of domain-specific experiences overall. Furthermore, the profile of cluster 1 consisted of a slightly above average amount in activities besides football (1969 h), which led to the highest sum of 7531 h for all learning activities together.

The second cluster showed *Club players* (*n* = 56) to have the earliest initial club participation (5.4 years) and subsequently the highest amount of in-club practice (1602 h). Within free football, these *Club players* had a lower number of around 2270 h compared to the Football enthusiasts and, even in activities besides football, they were slightly below average (1737 h). Overall cluster 2 reached an amount of 5328 h within all learning activities.

The largest group of *Average players* (*n* = 106) without special characteristics, i.e., *z*-scores < |0.5| in each operating factor, was found in cluster 3. They completed a total number of 4127 h in the three sections of learning activities (1072 h in-club practice, 1695 h free play and 1360 h activities besides football). Their highest absolute *z*-standardised value concerned the diversified sports activities besides football, with around 500 h below average.

A group with an extraordinarily high engagement in sports activities besides football (3593 h) was found in cluster 4, which led to the description as *Poly-sportive players* (*n* = 42). As regards football-specific activities, these players showed a slightly below average amount of in-club practice (1091 h) and an above average amount of free play within football (2359 hrs), while they started their careers in football clubs at an average age (6.4 years). Overall, this cluster completed 7043 h in the three learning activities.

Finally, *Football abstainers* (*n* = 61) formed cluster 5. These players started later in the organised football than their peers (7.9 years) and they did not participate that much in domain-specific (743 h in-club practice and 1645 h free play) or diversified activities (1493 h), whereby they only reached a total amount of 3881 h.

Regarding the transition analysis from clusters to youth football success levels, several significant differences from the expected values arose. First, *Football abstainers* showed an increased probability of playing football only on a local level (*OR* [95% CI] = 5.6 [2.9; 10.6], *χ*^2^(1) = 19.79, *p* < 0.01) and reduced chances for higher youth football success levels, as players from this cluster could rarely move up to a regional (*OR* = 0.5 [0.3; 0.9], *χ*^2^(1) = 1.63, *p* = 0.02) or national squad (*OR* = 0.3 [0.1; 0.8], *χ*^2^(1) = 4.07, *p* = 0.01). Analysing the transitions of *Poly-sportive players*, there was an above average chance for them to become regional players (*OR* = 2.2 [1.0; 4.6], *χ*^2^(1) = 1.35, *p* = 0.03). Furthermore, players from this cluster had a reduced risk of ending up in local level football (*OR* = 0.3 [0.1; 1.0], *χ*^2^(1) = 2.85, *p* = 0.03). The transitions of *Average players* from cluster 3 did not show any differences from expected values, while cluster 2 and its *Club players* were almost completely absent from the least successful local level (*OR* = 0.1 [0.1; 0.5], *χ*^2^(1) = 8.33, *p* < 0.01). In contrast, the constellation in this cluster led to an increase in transitions to the national level (*OR* = 2.1 [1.1; 4.1], *χ*^2^(1) = 3.26, *p* = 0.02). Finally, the most successful cluster in terms of transitions to the SJNT was the *Football enthusiasts* (*OR* = 3.1 [1.3; 7.3], *χ*^2^(1) = 5.26, *p* = 0.01), which had also a reduced tendency to be at the average regional level (*OR* = 0.4 [0.2; 1.0], *χ*^2^(1) = 1.32, *p* = 0.04).

## Discussion

The presented findings support previous work showing the essential role of sport participation up to 12 years of age (Côté et al., 2007), as its characteristics significantly influence the chance to participate in a SJNT. *Football enthusiasts* and *Club players* have an increased chance of becoming selected for this highest level of youth football in Switzerland, whilst *Football abstainers* have hardly any. In between *Average players* distribute to youth football success levels as expected by chance, whilst *Poly-sportive players* tend to reach the average regional level most frequently.

### Domain Specificity

As regards the question of the dimension of domain specificity, these results indicate the crucial role of specialisation within early sport participation. The most successful clusters in terms of SJNT selections completed the highest amounts of domain-specific learning activities (in club-practice and free play together). *Football abstainers* had by far the lowest amount in this area. Of course, taking only these three clusters into account, one can also refer to the relevance of the amount of sporting activities at all, as in comparing *Football abstainers* (3881 h) with *Club players* (5328 h) and to *Football enthusiasts* (7531 h), the total number of hours in all of the three learning activities increases. Adding the *Poly-sportive players* (7043 h) to this analysis, the pattern changes and highlights the importance of the domain specificity of learning activities. A combination of these figures may lead to the conclusion that the chance of reaching higher levels of youth football increases from an early sport participation consisting of fewer learning activities (*Football abstainers*; tendency for local level), to one with less specific activities (*Average players* and *Poly-sportive players*; tendency for regional level) up to one with a high amount of domain-specific activities (*Football enthusiasts* and *Club players*; tendency for national level). In addition, this promising pathway of early specialisation goes in general along with an early entrance into the organised sport, as the two most successful clusters started their careers in football clubs at the earliest age (5.5 and 5.4 years). All of these findings seem to be in line with the early engagement hypothesis (Ford et al., 2009), which has been offered as a football-specific pathway to expertise before and has been supported by many other studies (Ford and Williams, 2012; Roca et al., 2012; Williams et al., 2012; Zibung and Conzelmann, 2013; Haugaasen et al., 2014).

Of course, the current operationalisation of domain specificity does not fulfil the need for more detailed analysis of diversified activities in the development of talented football players as claimed in previous studies (Williams et al., 2012; Coutinho et al., 2016b; Hornig et al., 2016). However, perceiving domain specificity as a continuum, the formation of discrete categories within learning activities apart from football (e.g., other team sports, racket games, and centimetres, gramme, or seconds sports) appeared to be somewhat inconsistent. Furthermore, the low amount of diversified learning activities recognised within comparable samples in the literature (Ford et al., 2009; Ford and Williams, 2012; Roca et al., 2012; Williams et al., 2012; Haugaasen et al., 2014; Hornig et al., 2016) seemed to be contradictory to an important role for developing expertise and a more detailed analysis of these activities. Finally methodological issues according to the LICUR method limit the number of involved operating factors (Bergman et al., 2003), whereby summing up all sporting activities besides football into a single operating factor was the corollary. In summary, there was a substantial number of hours within this factor (1837 h; 36.6% of all learning activities), which nevertheless may have less influence on the outcome according to youth football success levels than domain-specific activities have.

### Performance Orientation

The role of performance orientation within early sports participation was a second issue of interest. The results regarding this dimension seem ambiguous. *Football enthusiasts* and *Club players* showed completely different patterns according to the value of performance orientation within their domain-specific activities. *Club players* showed the highest amount of performance-oriented in-club practice and an average number of hours in fun-oriented free football. Vice versa, *Football enthusiasts* showed up as the most successful group by completing 2270 h more within free football, whilst missing around 300 h of in-club practice compared to the aforementioned *Club players*. Certainly, this superiority of *Football enthusiasts* can be interpreted in different ways. It seems clear that their higher total amount of learning activities may predict at least a part of this increased chance for being selected to SJNTs. An alternative assumption focuses on the characteristics of the less structured free play. Following Côté and Erickson (2015) this kind of learning activity without supervision may tend to take part within many different *settings* of the sport domain (e.g., playing beach soccer with friends, playing in a park after school, and playing on your own). Moreover, this tendency seems to induce some kind of domain-specific diversity. Subsequently Côté and Erickson (2015) connected this perception with issues of the dimension of domain specificity, leading to the mentioned change in their understanding of diversification/sampling. In this regard, they stated that this term may not only describe a sampling of different experiences from different sport domains (low amount of domain specificity; Côté et al., 2009), but also a high amount of domain specificity, if it is combined with participation within different settings of the same sport domain. In that case it may be possible to utilise positive effects of a diversification/sampling (e.g., transfer effects, increased intrinsic motivation; Fransen et al., 2012; Hendry et al., 2014; Vink et al., 2015), without losing domain-specific activity volume. In other words, this model would allow a concurrent specialisation and sampling, which may be expressed as *specialised sampling* ^1^. This could explain a positive effect of participating within diversified forms of domain-specific free play, and could further highlight the relevance of experiencing the whole scope of performance orientation within different learning activities of the same sport domain. This matches with the findings of the current investigation and the early engagement hypothesis (Ford et al., 2009); substantial amounts of both examined domain-specific settings, free play and in-club practice together, are promising for later success in football.

Similar to restrictions concerning domain specificity, some aspects of data collection confound the insights on performance orientation. First, the operationalisation through free play and in-club practice is feasible, but both categories are difficult to classify in terms of performance orientation. As just mentioned, free play may take part in many different settings, which also tend to differ in their level of performance orientation. Further, Helsen et al. (1998) already stated that the definition of deliberate practice, perceived as activity with the highest value of performance orientation, may be difficult in the context of team sports, since Ericsson et al. (1993) aligned their framework to individual practice. In conjunction with this, it seems to be questionable whether in-club practice during early sport participation, which may often take place in small clubs with limited resources (e.g., low number of well-educated coaches; Howie and Allison, 2015) and heterogeneous training groups (Helsen et al., 1998), can fulfil the criteria of a highly performance-oriented learning activity. Second, quality criteria for the learning activities (Ford et al., 2010b; Coutinho et al., 2016a), have not been recorded nor taken into account in the current investigation. Anyway, the stated relevance of a diversity within domain-specific activities induced by several degrees of performance orientation (i.e., specialised sampling) seems plausible, as many aforementioned results support this insight.

### General Limitations

Whenever humans have to think back and remember specific details of their past, forgetting and uncertainty is inevitable to a certain degree (Hopwood, 2015). Participants and their parents had to think back around 6.6 years on average to complete the retrospective questionnaire in the current investigation. Compared to many studies dealing with expertise on adult level (e.g., Zibung and Conzelmann, 2013; Hornig et al., 2016), this is a relatively short period. However, to the best of our knowledge no investigation about psychometric properties of retrospective questionnaires on that amount of time exists. Over shorter periods, common values for validity and reliability of involvement in practice activities (hours per week or per year) and age at initial participation in primary sport were presented in the methods section. Nevertheless, Pearson correlations might hide the problem of overestimation (Hodges and Starkes, 1996; Helsen et al., 1998). Whilst the ratio between participants is rather stable, the absolute number of estimated hours in sporting activities seem to increase with chronological distance to the estimation. Further, the accuracy of recall is influenced by the organisational structure, which means that more structured and regular activity types show superiority over free playing amounts (Memmert et al., 2010).

Up to now, there is no unified understanding how to operationalise early sport participation. Following a substantial number of contributions (Baker et al., 2003a; Moesch et al., 2011; Zibung and Conzelmann, 2013; Hendry et al., 2014; Coutinho et al., 2016a; Hornig et al., 2016), we used practice and play oriented activities in primary sport, training activities besides the primary domain and age at initial primary sport club participation. Extending this definition, accumulated amounts in competition have complemented this operationalisation many times to a relevant extent (Ford et al., 2009; Roca et al., 2012; Ford and Williams, 2017). However, as the Swiss football association tries to regulate the amount of competition in certain age groups, we assume that a smaller amount of information was left behind than in other environmental circumstances, where the number of competitions is up to each individual club.

Overall, impaired psychometric properties and leaving out some information within operationalisation of early sport participation limit presented results and may omit further insights (e.g., small groups with other specific development constellations) to a certain extent. On the other side, these limitations do not result from carelessness nor neglect. The applied method illustrates consensual thoughts on contributing to the most common promising pathways of early sport participation, which we balanced to the best of our knowledge.

## Conclusion

This study examined the influence of characteristics of learning activities within early sport participation on youth football success levels. Whilst Zibung and Conzelmann (2013) investigated SJNT players and their later success in senior football, the present research dealt with the most promising pathway to even reach the SJNT level from a broader sample. According to this, around 15 years between the researched cohorts and a shift to a different selection level within Swiss football did not lead to any fundamental changes in terms of arising patterns within cluster constellations. Thus, the current contribution together with that of Zibung and Conzelmann (2013) draw a coherent picture of the talent development system in Swiss football. To succeed within this system, a huge amount of domain-specific learning activities within early sport participation is recommended.

**FIGURE 3:**
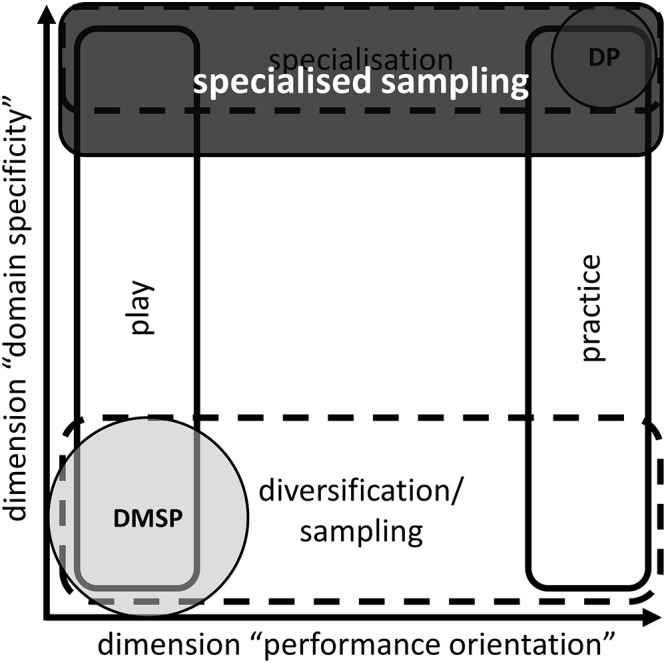
The specialised sampling model within the two-dimensional construct of domain specificity and performance orientation in relation to deliberate practice framework (DP) and the elite performance through sampling pathway from the development model of sports participation (DMSP).

However, we definitely do not claim that our data support the deliberate practice framework (Ericsson et al., 1993). Quite the contrary, the most successful cluster of *Football enthusiasts* with its extraordinary amount of free play supports the hypothesis that a broad range of settings within domain-specific learning activities may lead to superior success later in the football career. These diversified settings include different values of performance orientation (e.g., free play and in-club activities) as well as miscellaneous activities (e.g., beach soccer, playing in a park, and organised match play). Following the concept of Côté and Erickson (2015), who described this as a new understanding of diversification/sampling (i.e., sampling different settings within one domain instead of sampling between several domains), based on our data we perceive the talent development in Swiss football as a *specialised sampling model* (cf. **Figure 3**).

Nevertheless, mechanisms behind the specialised sampling model remain unclear to a certain extent. Of course, hypothesis of sampling/diversification pathways have been suggested earlier (e.g., transfer effects, increased intrinsic motivation; Fransen et al., 2012; Hendry et al., 2014; Vink et al., 2015). In that case, specialised sampling may reflect some kind of seeking for diversity. Depending on the environmental context, there may be different solutions to do this (Storm et al., 2012; Suppiah et al., 2015). Although Switzerland has a reasonable multi-sport history (e.g., winning Olympic diploma in 20 different sport domains in last summer and winter games) and multi-sport ideology in terms of national coaches education and public funding, football plays a key role in Swiss youth sport participation. The success of the senior national team (e.g., placing fourth in FIFA nations ranking in august 2017) and the high number of local clubs and registered players (Romann and Fuchslocher, 2013) result in a major impact on general society. Therefore, the search for diversity in Swiss youth sport participation may result in different settings of football as the most attractive solution. Further, supporting mechanisms of specialised sampling may connect to deliberate practice framework contents. Its deliberate practice activities are by itself not inherently enjoyable (Ericsson et al., 1993), whilst enjoyment of activities has been described as essential for keeping intrinsic motivation and following goals sustainable (Hendry et al., 2014). From that point of view, sampling several activities within the domain may reflect an addition of more enjoyable activities to keep the less enjoyable, more performance-oriented activities going. In addition, deliberate practice framework already stated on the embedment of playful activities for recovery (Ericsson et al., 1993). In that case, some amount of free play may have value for an active, biologocial and/or psychological recovery (Saw et al., 2016) from more intense and structured in-club practice.

Overall, it is very complex to unfold the exact mechanisms of successful patterns of early sport participation completely. Different cultures (e.g., availability of diversity of sport domains, popularity of a certain domain) and structures (e.g., talent development programmes) may significantly influence the success of certain development pathways (Storm et al., 2012; Suppiah et al., 2015). In Swiss football, we assume a specialised sampling model with a high degree of domain specificity within early sport participation (specialisation), which is enriched by a sport-specific diversity resulting from a broad range of settings within football (sampling), to be the most promising one.

If the existence of such models may be further supported by subsequent research, this could lead to a resolution of the specialise or sample debate (Côté et al., 2009). The ideas of former opposite counterparts would merge somewhere in the middle of extreme positions by enabling a coexistence of specialising and sampling through the specialised sampling model.

## Author Contributions

Substantial contributions to the conception or design of the work, interpretation of data, drafting the work or revising it critically for important intellectual content, final approval of the version to be published, and agreement to be accountable for all aspects of the work in ensuring that questions related to the accuracy or integrity of any part of the work are appropriately investigated and resolved: RS, CZ, MZ, and AC. Data acquisition: CZ. Data analysis: RS and MZ.

## Conflict of Interest Statement

The authors declare that the research was conducted in the absence of any commercial or financial relationships that could be construed as a potential conflict of interest.

## Footnotes

1 *Specialised* in terms of *specialised sampling* is close to the common understanding of specialisation (Wiersma, 2000) and addresses a focus on the primary sport domain (high value of domain specificity). *Sampling*, as the second part of *specialised sampling* addresses the use of different kinds of activities (i.e., different settings with different values of performance orientation in the primary sport domain) for the explicit purpose of developing domain specific requirements within the primary sport domain (e.g., perceptual-cognitive skills or physiological capabilities). Compared to the original understanding of *sampling* (Côté et al., 2007), this seems to be only part of the intended message. In this context, *specialised sampling* does not address further psychosocial-oriented implications of *sampling*.
